# Estimation of Genetic Parameters for Egg Production and Clutch Traits in Lindian Chickens

**DOI:** 10.3390/ani15131867

**Published:** 2025-06-24

**Authors:** Jiacheng Liu, Fei Liang, Changsheng Sun, Xu Wang, Zhiyong Su, Yumao Li, Peng Luan, Zhiping Cao, Xue Bai, Li Leng

**Affiliations:** 1 Key Laboratory of Chicken Genetics and Breeding, Ministry of Agriculture and Rural Affairs, Harbin 150030, China; l2631165626@163.com (J.L.); lf19971109@163.com (F.L.); 17513377630@163.com (C.S.); 18836934247@163.com (X.W.); su_zhiyong@neau.edu.cn (Z.S.); liyumao@neau.edu.cn (Y.L.); luan0901@neau.edu.cn (P.L.); caozhiping@neau.edu.cn (Z.C.); xuebai@neau.edu.cn (X.B.); 2 Key Laboratory of Animal Genetics, Breeding and Reproduction, Education Department of Heilongjiang Province, Harbin 150030, China; 3 College of Animal Science and Technology, Northeast Agricultural University, Harbin 150030, China

**Keywords:** Lindian chicken, egg production, genetic parameter, clutch, age at first egg

## Abstract

To accelerate breeding progress for egg production traits in Lindian chickens, the genetic parameters for egg production and clutch-related traits in Lindian chickens were evaluated in the present study. Data regarding the age at first egg (AFE), egg number (EN), average clutch length (ACL), and average pause length (APL) were collected from two generations of Lindian chickens based on individual egg production records at 32 weeks of age (32–wk), 43–wk, and 52–wk. The results showed that the AFE of Lindian chickens was 179.3 d of age, with a heritability of 0.35. The heritability was 0.26 for EN32, 0.28 for EN43, and 0.34 for EN52. ACL showed moderate-to-high heritability (h^2^ = 0.3–0.54), but APL traits showed low heritability (h^2^ = 0.09–0.14). There were high positive genetic and phenotypic correlations for EN in the three periods from the start of laying up to 32–wk, 43–wk, and 52–wk. EN had high negative genetic correlations with AFE (r_G_ = −0.47–−0.80) and high positive genetic correlations with ACL (r_G_ = 0.45–0.81). The correlation between EN and APL was positive for 32–wk and 43–wk, but negative for 52–wk. These results indicated that the egg production of Lindian chickens could be improved by the selection of AFE, early EN, and ACL.

## Simple Summary

Lindian chicken is a local breed in northern China. To accelerate the breeding progress for egg production traits in Lindian chickens, this study evaluated their egg production and clutch-related traits. The results indicated that the egg production of Lindian chickens could be improved by selecting for age at first egg (AFE), early egg number (EN), and average clutch length (ACL).

## 1. Introduction

China is the leading country in egg production, contributing over 35% of the total global output [1,2]. In China, around 10% of eggs are sourced from indigenous dual-purpose chicken breeds. Among these breeds, the Lindian chicken, a Chinese local breed, is famous for the unique texture and flavor of its meat and eggs, sharing comparable premium egg quality traits with specialized Chinese breeds like the blue-eggshell line [3], and has been listed on the National Animal Genetic Resources Protection List of China since 2014. Lindian hens typically start laying at approximately 25 weeks of age (25–wk), yielding a total of 110 to 120 high-quality eggs over a production period of 52–wk. Because of their lower egg production, it is necessary to use selection strategies to improve production efficiency in pure Lindian chicken lines. Egg production is a complex trait which may be affected by many factors, such as age at first egg (AFE) and clutch traits [4,5,6]. Many studies have reported on the genetic parameter of AFE and its relationship with egg production traits [7,8,9,10], but studies about clutch traits, which are characteristics of individual egg-laying patterns, have only been investigated in recent years. The AFE of Thai native chickens was around 28−wk of age, with an estimated heritability of 0.16 [11], while the AFE of Beijing-You chickens is 174.45 d of age, exhibiting a higher heritability of 0.62 [12]. Previous studies found that the heritability for egg number (EN) was 0.26 (EN43) and 0.18 (EN66) [12]. For the clutch traits of Beijing-You chickens, average clutch length (ACL) was shown to have a moderate heritability (h^2^ = 0.35–0.38), whereas average pause length (APL) showed a markedly low heritability (h^2^ = 0.05). So far, work on the genetic parameters of egg production and related clutch traits in Lindian chickens has not been systematically reported.

The estimation of genetic parameters for these egg production and clutch-related traits is necessary in order to improve our understanding of genetic architecture and explore proper biological traits for the improvement of egg production [12]. In this study, two generations of Lindian chicken preservation flocks which were born in 2021 and 2022 were used. The data for the AFE, EN, ACL, and APL at the three periods of 32–wk, 43–wk, and 52–wk were collected. The objective of the present study was to analyze the relationship between EN and other egg production-related traits, AFE, and clutch traits. Our findings will provide a reference for developing an appropriate selection strategy aimed at improving egg production traits in Lindian chickens.

## 2. Materials and Methods

### 2.1. Experimental Population

The experimental materials used in this study were raised on the experimental poultry breeding farm of Northeast Agricultural University. The pedigree was used over two generations, including a total of 2391 birds from the Lindian chicken preservation flock, which were born in 2021 and 2022 and raised in the same environmental conditions. In this experiment, individuals were reared in single cages and fed regularly according to the feeding program for laying hens.

### 2.2. Trait Measurements

For each individual, egg production from the first egg until 52–wk of age was recorded with an IVYSUN 2D barcode scanner (Ivysun, Chengdu, China), and the data collection system was used to calculate the egg production-related traits. The egg production cycle of Lindian hens can be divided into 3 stages: the early laying period, from the beginning of laying until reaching a laying rate of 70% (up to 32 weeks of age); the peak laying period, with a laying rate of more than 70% (33–43 weeks of age); and the late laying period, with a laying rate of less than 70% (44–52 weeks of age). AFE was taken as the age of the hen when laying its first egg, and EN was the total number of eggs laid in a given period. ACL represented the average number of days per clutch for each hen, while APL indicated the average number of non-laying days between clutches. The values of EN, ACL, and APL based on the 32–wk record (EN32: cumulative egg number up to 32 weeks of age; ACL32: average clutch length up to 32 weeks of age; and APL32: average pause length up to 32 weeks of age), 43–wk record (EN43, ACL43, and APL43), and 52–wk record (EN52, ACL52, and APL52) were calculated.

### 2.3. Statistical Analysis

In this study, the genetic parameters of egg production and clutch-related traits in Lindian chickens were estimated with the Average Information Restricted Maximum Likelihood method using ASReml version 4.2 software. The statistical model is as follows:

Y_ij_ = μ + G_i_ + a_j_ + e_ij_ (Model)

where Y_ij_ is the individual trait observation; μ is the population trait mean; G_i_ is the fixed effect of generation; a_j_ is the random direct additive genetic effect of the individual; and e_ij_ is the random residual effect. The variance components of the traits were estimated by the AI-REML algorithm using ASReml version 4.2 software, and the genetic parameters of the traits were further calculated. A single-trait model was used to estimate heritability; bivariate analyses were performed to compute genetic correlations between combinations of traits. For the raw data, outliers distributed outside the “mean ± 3σ” were first eliminated. Next, the leverage points of the residuals of each observation were calculated, and no outliers were found. The normality assumption of the AI-REML algorithm was validated using Q-Q plots of residuals for each trait, with the results indicating that the model residuals largely followed a normal distribution without significant deviation. We calculated the fitting parameters of the trait fitting model, including log-likelihood and Akaike Information Criterion values (AIC), and the results showed that the model we built had a high fitting effect, supporting the reliability of our results.

## 3. Results

### 3.1. Phenotypic Characteristics of Egg Production and Clutch Traits

The descriptive statistics of all phenotypic traits in this study are summarized in Table 1. The average AFE was 179.3 d of age with a coefficient of variation (CV) of 6.17%. The average EN increased by 285.6% from the 32–wk to the 52–wk period. The ACL of the 32–wk egg production record was 3.47 d and decreased to 2.64 d by the 52–wk record, while the APL of the 32–wk egg production record was 1.453 d and increased slightly to 1.524 d by the 52–wk record. The coefficient of variation for EN32 was larger than those for EN43 and EN52. The CV for AFE was less than 10%, while the CVs for EN32, EN43, EN52, ACL, and APL were higher than 15% during the experimental period, indicating that there was a large phenotypic variation for these traits in Lindian chickens.

### 3.2. Estimates of Genetic Parameters

**Figure 1:**
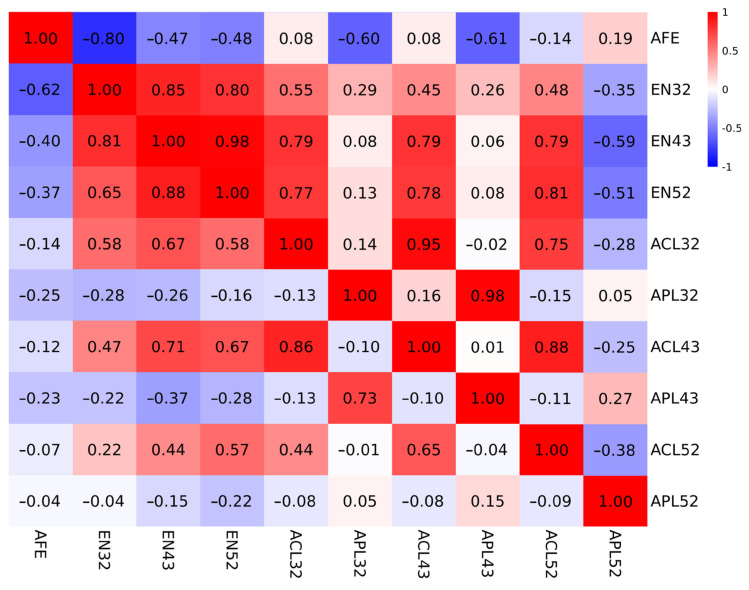
Heatmap of genetic (above the diagonal) and phenotypic (below the diagonal) correlations among clutch-related traits. The color gradient reflects correlation strength, with dark red (r = 1) and dark blue (r = −1) representing maximal positive and negative associations, respectively.

The heritability, genetic, and phenotypic correlations between egg production and clutch-related traits are shown in Table 2. The heritability values of EN during different periods in Lindian chickens in this study were medium (h^2^ = 0.26–0.34). It can be seen that as the laying period progresses, the heritability of egg production rates shows a slight upward trend. Figure 1 shows the correlation heatmaps for genetic and phenotypic correlations. The genetic and phenotypic correlations were high between EN values at each laying stage, indicating that early laying performance can effectively predict the egg production over the entire laying period. There were strong negative genetic (−0.47–−0.8) and phenotypic correlations (−0.37–−0.62) between AFE and EN during different periods. The EN had relatively higher correlations with ACL (r_G(EN32, ACL32)_ = 0.55; r_G(EN32, ACL43)_ = 0.45; r_G(EN32, ACL52)_ = 0.48; r_G(EN43, ACL32)_ = 0.79; r_G(EN43, ACL43)_ = 0.79; r_G(EN43, ACL52)_ = 0.79; r_G(EN52, ACL32)_ = 0.77; r_G(EN52, ACL43)_ = 0.78; r_G(EN52, ACL52)_ = 0.81), and negative correlations with APL52 (r_G(EN32, APL52)_ = −0.35; r_G(EN43, APL52)_ = −0.59; r_G(EN52, APL52)_ = −0.51). It was observed that EN32 had weak genetic correlations with APL32 and APL43 (r_G(EN32, APL32)_ = 0.29; r_G(EN32, APL42)_ = 0.26).

## 4. Discussion

As the standard of living improves, demands for high-quality egg products from indigenous chickens are growing. The Lindian chicken is a famous indigenous breed due to its outstanding egg quality in China; as such, improving its egg production is crucial in order to meet market demands. In this study, we estimated the genetic parameters for egg production and related traits in Lindian chickens, which can help us in the following selection practice.

### 4.1. AFE and Egg Production

Studies have indicated that the AFE significantly affects the reproductive performance of chickens: flocks with a normal AFE of 149–153 d exhibit optimal performance in terms of egg production, fertilization rate, and hormonal levels, while early-maturing (134–138 d) and late-maturing (>159 d) flocks demonstrate inferior reproductive performance [13]. A previous study on Rhode Island Red hens found that the phenotypic coefficients of variation were 22.67% and 25.89% for egg laying at 23–wk of age and egg laying at 61–80–wk of age, respectively, which was higher than the other traits (2.27–17.27%) [14]. Similarly, another indigenous Chinese breed, the Beijing-You chicken, also exhibited later AFE (174.45 d) and shorter ACL (3.53 d) at the 43–wk period [15]. Reports showed that the White Leghorn, Rhode Island Red, Columbian Plymouth Rock, Barred Plymouth Rock, synthetic dwarf line, and Thai Native Synthetic chicken breeds have AFE values of 144.26, 149.29, 147.5, 150.32, 154.61, and 168 d, respectively [15,16]. Compared with other breeds, Lindian chickens exhibit a relatively higher AFE and a lower ACL, suggesting that improving both parameters could enhance their egg production. The Lindian chicken is well known for its superior egg quality, and therefore improving its egg production is an important goal for breeding. Egg production largely depends on EN; the estimates of heritability for cumulative egg production in broiler chickens ranged from 0.16 to 0.54 between 1–wk and 40–wk of laying [17], which is consistent with the medium heritability (h^2^ = 0.26–0.34) of EN for Lindian chickens shown in this study.

AFE serves as a crucial indicator of sexual maturity in poultry, with its variation influenced by both genetic and environmental factors. Our study demonstrated moderate heritability for AFE in Lindian chickens (h^2^ = 0.35 ± 0.07), which aligns with the range (0.22–0.62) reported in previous studies [12,14,18,19]. Genetic correlation analysis revealed strong negative correlations between AFE and egg production across different stages (r_G_ = −0.80 with EN32; r_G_ = −0.47 with EN43; r_G_ = −0.48 with EN52; Table 2). This pattern suggests that alleles promoting earlier maturation may simultaneously enhance egg production, which closely matches findings in Beijing-You chickens [12]. The genetic correlations between AFE and egg production traits can provide critical guidance for selective breeding. Therefore, AFE can serve as a selection criterion for improving egg production performance in Lindian chickens.

### 4.2. ACL and Egg Production

The peak laying period and egg production cycle are also important factors in egg production. Indigenous breeds without selection show lower egg-laying peaks and maintenance. Clutch traits can be used to represent the rhythm of egg production, and by evaluating these traits, they can be applied in breeding practice. In our study, the heritability of ACL ranged from 0.3 to 0.54 across different laying stages in Lindian chickens. The high heritability of ACL implies strong genetic control over ovulatory cycles. In contrast, APL’s low heritability suggests it is primarily driven by environmental factors like temporary stress or disease. Previous studies found that the heritability of ACL is 0.35–0.38 for Beijing-You chickens and 0.23 for highly productive Rhode Island White hens [12,19]. These results indicate that indigenous breeds have higher genetic variation for ACL and thus could be improved significantly by selection. In Lindian chickens, the genetic correlations between ACL and EN were consistently high (r_G(EN32, ACL32)_ = 0.55; r_G(EN43, ACL43)_ = 0.79; r_G(EN52, ACL52)_ = 0.81), further supporting the idea that longer ACL is associated with higher egg production, and that selecting for ACL could improve egg production. Our results were consistent with previous reports of a positive correlation between ACL and EN [20]. This consistency across species, from turkeys to indigenous chickens [21], strengthens the biological plausibility of ACL as a universal selection marker for egg production. Therefore, selecting for longer ACL in Lindian chickens could be a feasible strategy for enhancing their overall egg production performance.

### 4.3. APL and Egg Production

APL showed low heritability (h^2^ = 0.05) and a negative genetic correlation with EN (r = −0.87 to −0.61) [15,20,22], indicating its limited suitability as a selection criterion for improving egg production. In Lindian chickens, APL also exhibited low heritability (h^2^ = 0.09–0.14) and was negatively correlated with EN (r_G(EN32, APL52)_ = −0.35; r_G(EN43, APL52)_ = −0.59; r_G(EN52, APL52)_ = −0.51). However, inconsistencies were found between the genetic and phenotypic correlations for EN and APL32 and APL43, while positive genetic correlations were found between EN and APL32 and APL43 (r_G_ = 0.12–0.28) with substantial standard errors (SE = 0.15–0.21). The phenotypic correlations between EN and APL were negative with smaller standard errors (r_P_ = −0.08 to −0.23; SE = 0.03–0.05). Given the low heritability and inconstancy of genetic and phenotypic correlations between EN and APL traits, APL is not a recommended trait for selection in breeding programs aiming to improve the egg-laying performance of Lindian chickens.

## 5. Conclusions

In summary, based on our results, AFE and ACL can simultaneously be used as selection criteria in breeding. Considering the high genetic correlations between EN32 and EN52 (r_G(EN32, EN52)_ = 0.80) and ACL32 and ACL52 (r_G(ACL32, ACL52)_ = 0.75), selection based on early egg production traits (EN32) and early ACL (ACL32) can be employed to improve their overall egg production and accelerate breeding progress in Lindian chickens. Our findings offer a valuable reference for breeding strategies aiming to improve the egg production of Lindian chickens.

## Figures and Tables

**Table 1:** Descriptive statistics of egg production and clutch traits in Lindian chickens.

Traits	N	Mean	SD	CV (%)	Maximum	Minimum
AFE	1413	179.30	9.82	5.48%	215	156
EN32	1413	29.13	7.48	25.68%	51	10
EN43	1413	78.25	12.91	16.50%	113	36
EN52	1413	112.30	20.85	18.56%	169	52
ACL32	1412	3.47	1.24	35.71%	10	1.69
APL32	1413	1.63	0.60	36.90%	5	1
ACL43	1403	3.22	0.99	30.68%	10	1.73
APL43	1407	1.45	0.37	25.41%	4.90	1
ACL52	1307	2.64	0.77	28.97%	9.80	1
APL52	1316	1.52	0.43	27.91%	5	1

Note: Maximum = Maximum value; Minimum = Minimum value.

**Table 2:** Heritability, phenotypic, and genetic correlations for egg production and clutch traits.

Traits	AFE	EN32	EN43	EN52	ACL32	APL32	ACL43	APL43	ACL52	APL52
AFE	**0.35 (0.07)**	−0.80 (0.07)	−0.47 (0.12)	−0.48 (0.12)	0.08 (0.15)	−0.60 (0.2)	0.08 (0.13)	−0.61 (0.19)	−0.14 (0.13)	0.19 (0.21)
EN32	−0.62 (0.02)	**0.26 (0.06)**	0.85 (0.05)	0.80 (0.07)	0.55 (0.11)	0.29 (0.28)	0.45 (0.11)	0.26 (0.26)	0.48 (0.12)	−0.35 (0.22)
EN43	−0.40 (0.02)	0.81 (0.01)	**0.28 (0.06)**	0.98 (0.02)	0.79 (0.07)	0.08 (0.25)	0.79 (0.06)	0.06 (0.25)	0.79 (0.08)	−0.59 (0.21)
EN52	−0.37 (0.02)	0.65 (0.02)	0.88 (0.01)	**0.34 (0.07)**	0.77 (0.08)	0.13 (0.24)	0.78 (0.06)	0.08 (0.23)	0.81 (0.06)	−0.51 (0.2)
ACL32	−0.14 (0.03)	0.58 (0.02)	0.67 (0.02)	0.58 (0.02)	**0.30 (0.06)**	0.14 (0.23)	0.95 (0.02)	−0.02 (0.23)	0.75 (0.09)	−0.28 (0.21)
APL32	−0.25 (0.03)	−0.28 (0.03)	−0.26 (0.03)	−0.16 (0.03)	−0.13 (0.03)	**0.09 (0.05)**	0.16 (0.2)	0.98 (0.11)	−0.15 (0.21)	0.05 (0.31)
ACL43	−0.12 (0.03)	0.47 (0.02)	0.71 (0.01)	0.67 (0.02)	0.86 (0.01)	−0.10 (0.03)	**0.52 (0.07)**	0.01 (0.2)	0.88 (0.04)	−0.25 (0.18)
APL43	−0.23 (0.03)	−0.22 (0.03)	−0.37 (0.02)	−0.28 (0.03)	−0.13 (0.03)	0.73 (0.01)	−0.10 (0.03)	**0.10 (0.05)**	−0.11 (0.2)	0.27 (0.29)
ACL52	−0.07 (0.03)	0.22 (0.03)	0.44 (0.02)	0.57 (0.02)	0.44 (0.02)	−0.01 (0.03)	0.65 (0.02)	−0.04 (0.03)	**0.54 (0.07)**	−0.38 (0.18)
APL52	−0.04 (0.03)	−0.04 (0.03)	−0.15 (0.03)	−0.22 (0.03)	−0.08 (0.03)	0.05 (0.03)	−0.08 (0.03)	0.15 (0.03)	−0.09 (0.03)	**0.14 (0.05)**

Note: The diagonal in bold is the heritability of the trait; genetic correlations are above the diagonal, and phenotypic correlations are below the diagonal, with standard errors in parentheses.

## Data Availability

The original contributions presented in this study are included in the article/Supplementary Materials. Further inquiries can be directed to the corresponding author.

## Supplementary Materials

The following supporting information can be downloaded at: https://www.mdpi.com/article/10.3390/ani15131867/s1. Figure S1: Q-Q plot (EN32). A Q-Q plot comparing the theoretical quantitative values of the residuals (x-axis) with the sample quantitative values (y-axis). Points along the diagonal indicate normality, while deviations indicate skewness or heavy tailing. File S1: Leverage points for traits (columns: HatMatrix, HatDiag, and LeveragePoint). File S2: Comparison of log-likelihood and AIC values for models with generation fixed effects vs. models without fixed effects. File S3: Descriptive statistics and estimation of genetic parameters for two generational traits in Lindian chickens.
