# Supplementary material for: Estimation of Genetic Parameters for Egg Production and Clutch Traits in Lindian Chickens
Source: Animals (Basel). 2025 Jun 24;15(13):1867. doi: 10.3390/ani15131867 (PMC12248892; doi:10.3390/ani15131867)
Supplement: Supplementary file 1 [file animals-15-01867-s001.zip › File S3.pdf]

Table S1. Descriptive statistics of egg production and clutch traits in first-generation Lindian chickens.

Traits	**N**	**Mean**	**SD**	**CV (%)**	**Maximum**	**Minimum**
AFE	593	184.77	4.39	2.38%	215	182
EN32	593	28.32	5.75	20.29%	42	10
EN43	593	75.70	11.22	14.82%	106	42
EN52	593	104.05	18.31	17.60%	150	52
ACL32	593	3.34	1.22	36.57%	10	1.69
APL32	593	1.43	0.35	24.84%	3.5	1
ACL43	583	3.07	0.92	29.82%	10	1.73
APL43	587	1.46	0.44	30.38%	4.89	1
ACL52	501	2.62	0.78	29.63%	7.75	1
APL52	500	1.69	0.51	30.14%	5	1

Note: Maximum = Maximum value; Minimum = Minimum value.

Table S2. Descriptive statistics of egg production and clutch traits in second-generation Lindian chickens.

Traits	**N**	**Mean**	**SD**	**CV (%)**	**Maximum**	**Minimum**
AFE	820	175.34	10.71	6.11%	210	156
EN32	820	29.72	8.47	28.50%	51	10
EN43	820	80.10	13.72	17.13%	113	36
EN52	820	118.27	20.54	17.37%	169	56
ACL32	819	3.56	1.24	34.91%	10	2
APL32	820	1.78	0.69	38.99%	5	1
ACL43	820	3.33	1.02	30.77%	8.9	2
APL43	820	1.45	0.30	21.04%	3.1	1
ACL52	806	2.65	0.76	28.57%	9.8	2
APL52	816	1.42	0.32	22.73%	4.2	1

Note: Maximum = Maximum value; Minimum = Minimum value

Table S3. Heritability, phenotypic, and genetic correlations for egg production and clutch traits in first-generation Lindian chickens.

Traits	**AFE**	**EN32**	**EN43**	**EN52**	**ACL32**	**APL32**	**ACL43**	**APL43**	**ACL52**	**APL52**
AFE	0.03 (0.07)	−0.92 (0.50)	−0.82 (0.72)	−0.94 (0.56)	0.04 (0.75)	−0.80 (0.85)	−0.26 (0.61)	−0.91 (0.99)	−0.35 (0.73)	0.60 (1.21)
EN32	−0.56 (0.03)	0.05 (0.07)	0.91 (0.27)	0.93 (0.34)	0.44 (0.41)	−0.97 (0.69)	0.64 (0.35)	−0.95 (0.91)	0.55 (0.48)	−0.46 (1.25)
EN43	−0.31 (0.04)	0.77 (0.02)	0.17 (0.09)	0.99 (0.07)	0.69 (0.19)	−0.50 (0.47)	0.82 (0.11)	−0.10 (0.43)	0.85 (0.17)	−0.35 (0.83)
EN52	−0.21 (0.04)	0.52 (0.03)	0.83 (0.01)	0.24 (0.09)	0.83 (0.16)	−0.32 (0.47)	0.83 (0.10)	−0.04 (0.39)	0.88 (0.10)	0.26 (0.67)
ACL32	−0.19 (0.04)	0.71 (0.02)	0.69 (0.02)	0.52 (0.03)	0.19 (0.09)	0.34 (0.61)	0.96 (0.05)	0.44 (0.42)	0.89 (0.17)	−0.14 (0.76)
APL32	−0.05 (0.04)	−0.50 (0.03)	−0.41 (0.03)	−0.26 (0.04)	−0.28 (0.04)	0.06 (0.07)	0.20 (0.44)	0.79 (0.38)	0.03 (0.38)	0.69 (1.09)
ACL43	−0.12 (0.04)	0.55 (0.03)	0.75 (0.02)	0.65 (0.02)	0.84 (0.01)	−0.22 (0.04)	0.50 (0.11)	0.41 (0.34)	0.98 (0.05)	0.31 (0.54)
APL43	−0.09 (0.04)	−0.29 (0.04)	−0.51 (0.03)	−0.37 (0.04)	−0.19 (0.04)	0.53 (0.03)	−0.18 (0.04)	0.10 (0.08)	−0.10 (0.30)	0.73 (0.89)
ACL52	−0.01 (0.04)	0.23 (0.04)	0.47 (0.04)	0.57 (0.03)	0.40 (0.04)	−0.06 (0.05)	0.64 (0.03)	−0.14 (0.05)	0.72 (0.12)	−0.16 (0.57)
APL52	−0.06 (0.04)	−0.01 (0.05)	−0.05 (0.05)	0.01 (0.05)	0.04 (0.05)	0.06 (0.05)	0.02 (0.05)	0.21 (0.04)	0.04 (0.05)	0.04 (0.08)

Note: The diagonal is the heritability of the trait; genetic correlations are above the diagonal, and phenotypic correlations are below the diagonal, with standard errors in parentheses.

Table S4. Heritability, phenotypic, and genetic correlations for egg production and clutch traits in second-generation Lindian chickens.

Traits	**AFE**	**EN32**	**EN43**	**EN52**	**ACL32**	**APL32**	**ACL43**	**APL43**	**ACL52**	**APL52**
AFE	0.42 (0.09)	−0.80 (0.08)	−0.51 (0.14)	−0.52 (0.13)	0.09 (0.18)	−0.69 (0.21)	0.11 (0.16)	−0.82 (0.22)	−0.23 (0.17)	0.19 (0.22)
EN32	−0.65 (0.02)	0.35 (0.09)	0.88 (0.05)	0.82 (0.07)	0.58 (0.12)	0.42(0.30)	0.46 (0.13)	0.43 (0.32)	0.67 (0.14)	−0.34 (0.23)
EN43	−0.45 (0.03)	0.82 (0.01)	0.35 (0.08)	0.99 (0.01)	0.82 (0.08)	0.22 (0.30)	0.78 (0.07)	0.15 (0.31)	0.90 (0.09)	−0.67 (0.20)
EN52	−0.44 (0.03)	0.71 (0.02)	0.91 (0.01)	0.40 (0.09)	0.75 (0.09)	0.27 (0.28)	0.76 (0.07)	0.15 (0.28)	0.89 (0.07)	−0.74 (0.16)
ACL32	−0.14 (0.04)	0.54 (0.03)	0.66 (0.02)	0.62 (0.02)	0.38 (0.08)	0.27 (0.26)	0.95 (0.02)	−0.26 (0.26)	0.78 (0.10)	−0.35 (0.21)
APL32	−0.31 (0.03)	−0.20 (0.03)	−0.18 (0.03)	−0.11 (0.04)	−0.05 (0.04)	0.11 (0.06)	−0.26 (0.24)	0.88 (0.11)	−0.25 (0.28)	0.10 (0.33)
ACL43	−0.13 (0.04)	0.45 (0.03)	0.69 (0.02)	0.69 (0.02)	0.88 (0.01)	−0.02 (0.04)	0.53 (0.09)	−0.21 (0.25)	0.89 (0.06)	−0.47 (0.19)
APL43	−0.29 (0.03)	−0.19 (0.03)	−0.30 (0.03)	−0.22 (0.03)	−0.09 (0.04)	0.84 (0.01)	−0.05 (0.04)	0.11 (0.06)	−0.08 (0.28)	0.16 (0.33)
ACL52	−0.10 (0.04)	0.24 (0.04)	0.44 (0.03)	0.59 (0.02)	0.48 (0.03)	0.03 (0.04)	0.67 (0.02)	0.03 (0.04)	0.35 (0.08)	−0.64 (0.18)
APL52	−0.04 (0.04)	−0.06 (0.04)	−0.21 (0.03)	−0.36 (0.03)	−0.14 (0.04)	0.05 (0.04)	−0.16 (0.04)	0.11 (0.04)	−0.20 (0.04)	0.20 (0.07)

Note: The diagonal is the heritability of the trait; genetic correlations are above the diagonal, and phenotypic correlations are below the diagonal, with standard errors in parenthese
