# Supplementary material for: Estimation of Genetic Parameters for Egg Production and Clutch Traits in Lindian Chickens
Source: Animals (Basel). 2025 Jun 24;15(13):1867. doi: 10.3390/ani15131867 (PMC12248892; doi:10.3390/ani15131867)
Supplement: Supplementary file 1 [file animals-15-01867-s001.zip › Note S1.pdf]

Figure S1：Q-Q plot (EN32). A Q-Q plot comparing the theoretical quantitative values of the residuals (x-axis) with the sample quantitative values (y-axis). Points along the diagonal indicate normality, while deviations indicate skewness or heavy tailing.

File S1: Leverage points for traits (columns: HatMatrix, HatDiag, and LeveragePoint).

File S2: Comparison of log-likelihood and AIC values for models with generation fixed effects vs. models without fixed effects.

File S3: Descriptive statistics and estimation of genetic parameters for two generational traits in Lindian chickens.
