# Supplementary figures and images for: Estimation of Genetic Parameters for Egg Production and Clutch Traits in Lindian Chickens
Source: Animals (Basel). 2025 Jun 24;15(13):1867. doi: 10.3390/ani15131867 (PMC12248892; doi:10.3390/ani15131867)

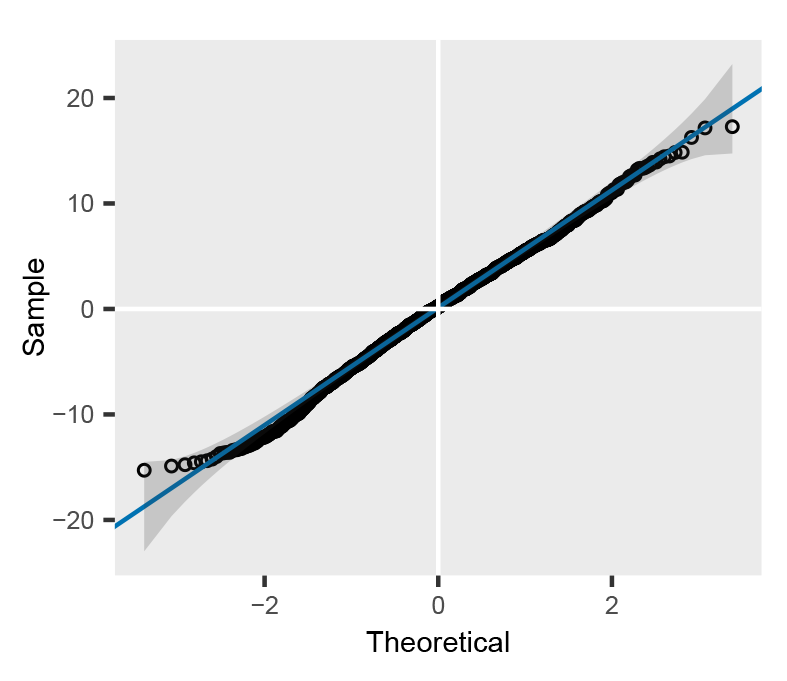

Supplement: Supplementary file 1 [file animals-15-01867-s001.zip › Figure S1.tif]
